# An analysis of trends and determinants of health insurance and healthcare utilisation in the Russian population between 2000 and 2004: the 'inverse care law' in action

**DOI:** 10.1186/1472-6963-9-68

**Published:** 2009-04-27

**Authors:** Francesca Perlman, Dina Balabanova, Martin McKee

**Affiliations:** 1 London School of Hygiene and Tropical Medicine, Keppel Street, London, WC1E 7HT, UK

## Abstract

**Background:**

The break-up of the USSR brought considerable disruption to health services in Russia. The uptake of compulsory health insurance rose rapidly after its introduction in 1993. However, by 2000 coverage was still incomplete, especially amongst the disadvantaged. By this time, however, the state health service had become more stable, and the private sector was growing. This paper describes subsequent trends and determinants of healthcare insurance coverage in Russia, and its relationship with health service utilisation, as well as the role of the private sector.

**Methods:**

Data were from the Russia Longitudinal Monitoring Survey, an annual household panel survey (2000–4) from 38 centres across the Russian Federation. Annual trends in insurance coverage were measured (2000–4). Cross-sectional multivariate analyses of the determinants of health insurance and its relationship with health care utilisation were performed in working-age people (18–59 years) using 2004 data.

**Results:**

Between 2000 and 2004, coverage by the compulsory insurance scheme increased from 88% to 94% of adults; however 10% of working-age men remained uninsured. Compulsory health insurance coverage was lower amongst the poor, unemployed, unhealthy and people outside the main cities. The uninsured were less likely to seek medical help for new health problems. 3% of respondents had supplementary (private) insurance, and rising utilisation of private healthcare was greatest amongst the more educated and wealthy.

**Conclusion:**

Despite high population insurance coverage, a multiply disadvantaged uninsured minority remains, with low utilisation of health services. Universal insurance could therefore increase access, and potentially contribute to reducing avoidable healthcare-related mortality. Meanwhile, the socioeconomically advantaged are turning increasingly to a growing private sector.

## Background

The Soviet Union created a health care system that was, at least officially, largely free at the point of use. However, after 1991, the newly independent states faced major challenges in maintaining this system, large parts of which had been linked to major employers that were becoming insolvent. [1] For many, access to health care became difficult, [2] with shortages of equipment and medication. [3] Although Russia may have coped better [4] than some of its neighbours, such as Kyrgyzstan [5] or the southern Caucasus [6] the 1990s were still a time of considerable difficulty. [3] Deaths preventable by timely and effective health care were much more common than in western countries. [7]

A system of statutory health care insurance was introduced in Russia in 1993 [3,8] and was intended to be universal and compulsory, but in reality it was not. Premiums were paid by employers, or in the case of the unemployed, retired and other vulnerable groups, by municipalities or regional authorities. This reflected a trend across central and eastern Europe and the former Soviet Union away from the former financing model based on general government revenues towards social insurance, primarily as a means of seeking to safeguard funds for health care from political interference, while maintaining the principle of universality. Implementation was initially uneven,[9] and often problematic. [10,11] Nevertheless, coverage increased rapidly amongst adults from an initial 3% to 88% in 2000; [12] however the uninsured minority consisted of the most disadvantaged: pensioners, the unemployed, people outside formal employment, and individuals residing outside Moscow and St Petersburg. [12]

The effects of health insurance and other determinants on health care utilisation in Russia have not been well researched. The first point of contact in Russia is often the Soviet-initiated polyclinic, and whilst in some cases these have been updated within the framework of implementation of a new model of integrated general practice, poor working conditions, infrastructure and financing mechanisms have hampered the process.[13]

Affordability is an important barrier to health care,[4,14] whilst education may also determine an individual's ability to negotiate the system. In one study in St Petersburg, the less educated perceived that they were helpless to overcome lengthy queues and apparently unsympathetic clinicians, in contrast with the strategic approach adopted by the more educated, who often used personal contacts to obtain the "best" care. [2,15,16] Unofficial payments to health professionals, often complemented by informal exchanges for services, may also benefit the better off, allowing them to receive better quality, more personal care [15,17]. These findings are consistent with the 'inverse health care law', described by Tudor Hart almost 40 years ago. This stated that "the availability of good medical care tends to vary inversely with the need for it in the population served", drawing on evidence that both the quantity and quality of care was worse in places such as the South Wales mining communities in which Tudor Hart worked than in more affluent parts of the United Kingdom. It has subsequently applied to all situations in which individuals in greatest need are least able to access effective care. [18]

The aims of this paper are first, to investigate trends and determinants of access to health insurance, updating earlier analyses, [12] and second, to study the effects of insurance and other variables on health care utilisation, both state and private, in transitional Russia after 2000.

## Methods

### Data

We used data from 5 study rounds (2000–4) of the Russia Longitudinal Monitoring Survey, a widely used panel survey of households and the individuals within them. Participants came from 38 population centres across the Russian Federation. St Petersburg and Moscow were selected automatically, and the remaining 36 districts, or primary sampling units (PSUs), were sampled by stratifying districts according to socioeconomic criteria, and selecting from each stratum using a probability proportional to size (PPS). Within the selected PSUs, urban and rural secondary sampling units (SSUs), census enumeration districts and villages respectively, were selected. From each SSU, 10 households were selected from housing lists developed by the investigators. The first dwelling was chosen at random, and the remainder at regular intervals. Thus, the sampling procedure was designed to achieve a study population that was broadly representative of the national population, but also ensuring that the two principal cities, with their particular characteristics, were included.

The overall response rate in the first round of Phase 2 (1994) was 84%, although it was lower in Moscow and St Petersburg (67%). In subsequent rounds, newly recruited households replaced those that left.

More details of the study methods are available at . Whilst the data are already publicly available, we obtained additional permission from the ethics committee at the London School of Hygiene and Tropical Medicine to conduct these analyses.

### Variables

#### (a) Health care variables

Respondents were asked first about "compulsory" or supplementary health insurance: "Do you have compulsory medical insurance, that is, a medical insurance policy?", and "Do you have supplementary voluntary medical insurance, with some form of service from an insurance firm, polyclinic, hospital, or medical centre?"

Regarding health service use, they were first asked "Have you in the last 30 days had any health problems?", and if the answer was yes, "What did you do to solve the health problems that you had in the last 30 days?". The two alternate responses were either seeking professional help (either at a medical institution or from a health worker), or treating oneself.

Respondents who had sought professional help for a health problem were asked "Let's talk about the most recent time you visited a medical worker in the last 30 days". The 6 possible responses were dichotomised into using the public sector (state polyclinic, state hospital or home visit) or the private sector (private polyclinic, hospital or physician).

#### (b) Socio-demographic variables

Respondents were defined as coming from an urban area, a rural area or the metropolitan areas (Moscow/St Petersburg). Marital status was divided into married/cohabiting, single (never married), divorced or widowed. Education was grouped into incomplete secondary or primary; complete secondary (technical, general or combined); and higher. Although other studies in the FSU region have disaggregated secondary vocational from secondary general (3), we found no significant differences between these two groups founding terms of the variables of interest. Household income per person was approximated by dividing total household income by the square root of the number of occupants. [19] An asset score was calculated by summing the number of selected consumer goods possessed (colour television, video recorder, car, washing machine, dacha [country cottage/hut with land for growing food]). Principal components analysis showed that these variables loaded onto a single factor, and could therefore be combined into a single continuous asset score.

### Statistical analysis

Overall trends were measured among respondents aged over 18. The prevalence of the access to health insurance and health care was measured in each year (2000–2004), using the above variables. The proportions were standardised to the age/sex structure of the population in the 2000 study round.

Multivariate logistic regression was used to study the determinants in working age respondents (aged 18–60) in the 2004 round, firstly of *not *having compulsory insurance (vs having insurance), and secondly of having private insurance cover (vs not having private insurance). 3 models were used (i) age and sex adjusted (ii) = (i) + income, education, area and marital status (iii) = (ii) + employment status. (However the analyses to identify any effect of asset score were not adjusted for income). Finally logistic regression was used to study the outcomes of seeking professional help for a health problem (vs not seeking), and using private health care (vs not using it). The effect of health insurance and a range of other predictor variables on these outcomes was studied. The same 3 multivariate models were used, and additionally the third model was adjusted for self-rated health.

## Results

Between 2000 and 2004, the proportion of the study population without compulsory health insurance declined from 12% to 6% (Table 1), however over 10% of working age men were still uninsured by 2004 (Table 1, Table 2).

**Table 1:** Coverage of health insurance and use of health care-RLMS 2000–2004 (respondents aged 18 and over)

		**Year**				
**Variable**		**2000**	**2001**	**2002**	**2003**	**2004**

**No of respondents** ***(mean age)***						
**Males**		3,534 *(43.0)*	3,902 *(42.9)*	4,074 *(42.9)*	4,137 *(42.4)*	4,571 *(39.7)*
**Females**		4,801 *(46.7)*	5,416 *(46.6)*	5,571 *(46.7)*	5,679 *(46.5)*	6,067 *(44.5)*

**COMPULSORY INSURANCE** **no (%)**						

**Whole sample**	**Y**	7,294 (87.9)	8,516 (91.5)	8,924 (92.7)	9,148 (93.4)	10,001 (94.1)
	**N**	1,008 (12.1)	788 (8.5)	708 (7.4)	648 (6.6)	623 (5.9)
***Subgroups***						
***Men <60 years***	***Y***	*2308 (82.4)*	*2,683 (86.6)*	*2861 (87.7)*	*2998 (88.9)*	*3068 (89.5)*
	***N***	*493 (17.6)*	*416 (13.4)*	*400 (12.3)*	*374 (11.1)*	*362 (10.6)*
***Women <60 years***	***Y***	*3003 (89.0)*	*3,531 (92.2)*	*3691 (93.3)*	*3874 (94.3)*	*3940 (94.8)*
	***N***	*371 (11.0)*	*298 (7.8)*	*266 (6.7)*	*234 (5.7)*	*216 (5.2)*
***Men >60 years***	***Y***	*677 (94.4)*	*768 (96.6)*	*793 (98.5)*	*739 (97.8)*	*695 (98.4)*
	***N***	*40 (5.6)*	*27 (3.4)*	*12 (1.5)*	*17 (2.3)*	*11 (1.6)*
***Women >60 years***	***Y***	*1306 (92.6)*	*1,534 (97.0)*	*1579 (98.1)*	*1537 (98.5)*	*1501 (98.9)*
	***N***	*104 (7.4)*	*47 (3.0)*	*30 (1.9)*	*23 (1.5)*	*17 (1.1)*

**SUPPLEMENTARY INSURANCE** **Whole sample – no. (%)**						
Yes		156 (1.9)	295 (3.2)	306 (3.2)	321 (3.3)	259 (2.5)
No		8,093 (98.1)	8,976 (96.8)	9,313 (96.8)	9,434 (96.7)	10,297 (97.6)

***Who pays for supplementary insurance – no. (%)***						
*Enterprise*		*112 (71.8)*	*225 (76.3)*	*227 (74.2)*	*252 (78.5)*	*202 (78.0)*
*Self*		*29 (18.6)*	*43 (14.6)*	*56 (18.3)*	*47 (14.6)*	*38 (14.7)*
*Other*		*15 (9.6)*	*27 (9.2)*	*23 (7.5)*	*22 (6.9)*	*19 (7.3)*

**SAW PROF. FOR NEW ILLNESS** **Whole sample – no. (%)**						
Yes		1,319 (37.1)	1,860 (29.5)	3,885 (69.5)	1,692 (31.3)	1,897 (32.4)
No		2,234 (62.9)	4,445 (70.5)	1,706 (30.5)	3,714 (68.7)	3,961 (67.6)

**PRIVATE HEALTH CARE** **Whole sample no. (%)**						
Yes		60 (5.8)	106 (7.0)	111 (7.9)	110 (8.2)	124 (8.0)
No		981 (94.2)	1,405 (93.0)	1,297 (92.1)	1,230 (91.8)	1,435 (92.1)

**Table 2:** Distribution of variables in people aged under 60 – 2004 round (for multivariate)

**Variable**	**No (%) of respondents**	
	**Male**	**Female**

**Total (by gender)**	3,864 (45.9)	4,549 (54.1)
**Education**		
Primary/incomplete 2ry	1,077 (27.9)	854 (18.8)
Complete secondary +/- technical	1,692 (43.9)	1,580 (34.8)
Higher	1,089 (28.2)	2,111 (46.5)
**Healthcare insurance**		
Compulsory	3,368 (87.3)	4,203 (92.5)
Supplementary (± compulsory)	125 (3.2)	118 (2.6)
None	363 (9.4)	223 (4.9)
**Use of private health care for problem**		
Yes	30 (6.0)	93 (8.8)
No	467 (94.0)	967 (91.2)
**Treated by**		
Dr	593 (29.0)	1,302 (34.2)
Self	1,454 (71.0)	2,501 (65.8)
**Marital status**		
Married	2,034 (52.7)	2,244 (49.4)
Single (never married)	1,140 (29.6)	1,049 (23.1)
Divorced, not remarried	203 (5.3)	469 (10.3)
Widowed	31 (0.8)	277 (6.1)
Cohabiting (not registered)	450 (11.7)	505 (11.1)
**Region of residence**		
Urban	2,217 (59.3)	2,637 (61.1)
Rural	1,004 (26.8)	1,053 (24.4)
Moscow/St. Petersburg	521 (13.9)	626 (14.5)
**Employment status**		
Currently working	2,476 (64.1)	2,652 (58.3)
Currently on paid leave	23 (0.6)	181 (4.0)
Currently on unpaid leave	2 (0.1)	14 (0.3)
Not working (other)	707 (18.3)	965 (21.2)
Unemployed (self-report)	469 (12.1)	368 (8.1)
Pensioner, not working	185 (4.8)	367 (8.1)
**Self-rated health**		
Very good	104 (2.7)	55 (1.2)
Good	1,671 (43.5)	1,412 (31.2)
Average	1,862 (48.5)	2,694 (59.5)
Poor	179 (4.7)	338 (7.5)
Very poor	24 (0.6)	30 (0.7)
**Some of household income comes from market economy (salary or investment)**		
Yes	1,939 (50.2)	2,392 (52.6)
No	1,925 (49.8)	2,157 (47.4)
Total	3,864 (100)	4,549 (100)
**Household income per person (income/sqrt no. household members) – Roubles **Mean (SD), *no of obs*	6219.7 (15114.1) *3690*	5763.4 (6081.4) *4226*

Characteristics of the 2004 round of the survey, used in the subsequent regression analyses, are shown in Table 2. Table 3 shows the determinants of not having compulsory insurance, adjusted according to the previously specified models. Women were less likely than men to be without cover. In both sexes, those in households with a higher asset score (consumer goods) were less likely to be uninsured. The effect of household income was similar in direction, but was largely explained by employment status in the multivariate analyses. The impact of marital status varied by gender, with single women significantly less likely, and cohabiting men significantly more likely, to lack cover than their married counterparts. Residents of Moscow and St. Petersburg were also significantly less likely than others to be uninsured. However, the most striking associations were with employment status. Those in receipt of pensions (including those below retirement age but suffering from disability or social problems) were very much less likely than the employed to be without cover, after adjustment for all other parameters whilst, in contrast, the unemployed were over three times more likely to be uninsured. Self-employed men were also less likely to lack cover, but for women this was not statistically significant, although the numbers involved were low. Worse self-rated health was independently associated with lacking statutory insurance.

**Table 3:** Determinants of *not *having compulsory medical insurance, and of having supplementary (private) insurance – results of logistic regression analyses (RLMS: 2004, 18–59)

	**Male**			**Female**		
	**(i) Age**	**(ii) Age, inc, educ, area, marital**	**(iii) = (ii) + employment status**	**(i) Age**	**(ii) Age, inc, educ, area, marital**	**(iii) = (ii) + employment status**
	***NOT HAVING COMPULSORY INSURANCE (vs having insurance) – Odds ratio (95% CI)***					

***Sex (female vs male)***	-	-	-	***0.49 (0.42–0.58)***	***0.46 (0.38–0.56)***	***0.47 (0.39–0.57)***

***Age (per year)***	*1.00 (0.99–1.01)*	*0.99 (0.98–1.00)*	*0.99 (0.97–1.00)*	*1.00 (0.99–1.01)*	*0.98 (0.97–1.00)*	*1.00 (0.98–1.01)*
***Education***						
*1y/incomp 2ry*	*1*	*1*	*1*	*1*	*1*	*1*
*Comp 2ry*	*1.05 (0.81–1.36)*	*1.02 (0.77–1.34)*	*0.99 (0.74–1.31)*	*0.97 (0.67–1.40)*	*0.93 (0.63–1.39)*	*0.88 (0.59–1.31)*
*Higher*	*0.89 (0.66–1.21)*	*1.04 (0.74–1.45)*	*1.03 (0.73–1.45)*	*0.78 (0.54–1.13)*	*0.76 (0.51–1.15)*	*0.79 (0.51–1.20)*
***Hhld inc. pp quintile (asc.)***	***0.85 (0.79–0.92)***	***0.90 (0.83–0.98)***	*0.97 (0.89–1.07)*	***0.86 (0.78–0.95)***	*0.90 (0.81–1.00)*	*0.94 (0.84–1.05)*
***Nr consumer gds (0–5)***	***0.80 (0.74–0.86)***	***0.80 (0.73–0.88)***	***0.86 (0.78–0.94)***	***0.85 (0.77–0.93)***	***0.84 (0.75–0.95)***	***0.86 (0.76–0.97)***
***Marital status***						
*Married*	*1*	*1*	*1*	*1*	*1*	*1*
*Single*	*0.72 (0.51–1.02)*	*0.73 (0.51–1.05)*	*0.74 (0.51–1.09)*	***0.48 (0.30–0.76)***	***0.42 (0.26–0.69)***	***0.41 (0.25–0.68)***
*Divorced*	***1.73 (1.12–2.67)***	*1.47 (0.92–2.33)*	*1.33 (0.82–2.15)*	*1.26 (0.82–1.95)*	*1.17 (0.75–1.85)*	*1.23 (0.77–1.95)*
*Widowed*	***3.03 (1.21–7.54)***	*1.90 (0.64–5.62)*	*2.12 (0.68–6.59)*	*1.63 (0.95–2.80)*	*1.29 (0.72–2.32)*	*1.41 (0.78–2.56)*
*Cohabiting*	***2.43 (1.81–3.24)***	***2.40 (1.78–3.24)***	***2.28 (1.68–3.10)***	***1.57 (1.07–2.30)***	*1.48 (0.99–2.22)*	*1.44 (0.95–2.18)*
***Area of residence***						
*Urban*	*1*	*1*	*1*	*1*	*1*	*1*
*Rural*	*1.19 (0.94–1.51)*	*1.17 (0.91–1.50)*	*0.93 (0.71–1.21)*	*0.99 (0.72–1.37)*	*0.90 (0.64–1.26)*	*0.82 (0.58–1.15)*
*Moscow/St P*	***0.40 (0.26–0.63)***	***0.46 (0.29–0.75)***	***0.49 (0.30–0.80)***	***0.55 (0.33–0.90)***	*0.61 (0.35–1.06)*	***0.56 (0.32–0.98)***
***Employment status***						
*Working*	*1*	*1*	*1*	*1*	*1*	*1*
*Paid leave*	*0.50 (0.07–3.71)*	*0.66 (0.52–0.84)*	*0.75 (0.58–0.97)*	*0.75 (0.30–1.88)*	*0.39 (0.12–1.28)*	*0.40 (0.12–1.28)*
*Not working*	*0.76 (0.53–1.10)*	*0.83 (0.54–1.27)*	*0.79 (0.52–1.21)*	***1.67 (1.16–2.42)***	***1.62 (1.08–2.46)***	***1.64 (1.08–2.48)***
*Unemployed (sr)*	***3.45 (2.66–4.45)***	***3.29 (2.47–4.40)***	***3.05 (2.27–4.10)***	***3.51 (2.43–5.08)***	***3.15 (2.12–4.68)***	***3.18 (2.13–4.75)***
*Pension*	***0.19 (0.06–0.59)***	***0.15 (0.04–0.61)***	***0.14 (0.03–0.57)***	***0.28 (0.11–0.69)***	***0.33 (0.13–0.83)***	***0.33 (0.13–0.84)***
***Self-rated hlth (vg -5(vp)***	***1.32 (1.11–1.56)***	***1.30 (1.09–1.56)***	***1.41 (1.17–1.70)***	*1.10 (0.88–1.38)*	*1.08 (0.85–1.37)*	*1.17 (0.91–1.49)*

	***HAVING SUPPLEMENTARY (PRIVATE) INSURANCE (vs not having) Odds ratio (95% CI)***					

***Sex (female vs male)***	-	-	-	*0.77 (0.60–1.00)*	***0.70 (0.53–.093)***	*0.75 (0.56–1.00)*

***Age (per year)***	***1.03 (1.02–1.04)***	*1.01 (0.99–1.03)*	*1.01 (0.99–1.03)*	*1.02 (1.00–1.03)*	*1.02 (1.00–1.04)*	*1.02 (1.00–1.04)*
***Education***						
*1y/incomp 2ry*	*1*	*1*	*1*	*1*	*1*	*1*
*Comp 2ry*	***3.41 (1.73–6.72)***	***2.54 (1.27–5.09)***	***2.30 (1.15–4.59)***	***3.43 (1.45–8.15)***	*2.22 (0.91–5.44)*	*1.99 (0.81–4.90)*
*Higher*	***4.90 (2.46–9.75)***	***2.49 (1.22–5.07)***	***2.05 (1.01–4.16)***	***4.68 (2.01–10.87)***	***2.62 (1.10–6.27)***	*1.93 (0.80–4.65)*
***Hhld inc. pp quintile (asc.)***	***1.88 (1.58–2.24)***	***1.76 (1.46–2.12)***	***1.73 (1.43–2.09)***	***1.88 (1.56–2.25)***	***1.70 (1.40–2.07)***	***1.65 (1.35–2.02)***
***Nr consumer gds (0–5)***	*1.26 (1.09–1.46)*	*1.14 (0.97–1.34)*	*1.13 (0.96–1.34)*	***1.19 (1.03–1.37)***	***1.31 (1.10–1.55)***	***1.32 (1.10–1.58)***
***Marital status***						
*Married*	*1*	*1*	*1*	*1*	*1*	*1*
*Single*	***0.31 (0.16–0.61)***	***0.38 (0.19–0.76)***	*0.63 (0.32–1.27)*	*0.98 (0.55–1.74)*	*1.25 (0.68–2.32)*	*1.62 (0.88–2.98)*
*Divorced*	***0.30 (0.10–0.96)***	*0.44 (0.14–1.43)*	*0.47 (0.14–1.52)*	*1.11 (0.64–1.94)*	*1.21 (0.64–2.27)*	*1.11 (0.59–2.12)*
*Widowed*	*0.61 (0.08–4.52)*	*0.81 (0.10–6.32)*	*0.99 (0.12–8.07)*	*0.40 (0.14–1.13)*	*0.69 (0.24–1.97)*	*0.70 (0.24–2.02)*
*Cohabiting*	*0.53 (0.28–1.01)*	*0.58 (0.30–1.13)*	*0.61 (0.31–1.17)*	*0.73 (0.37–1.44)*	*0.67 (0.30–1.51)*	*0.69 (0.31–1.56)*
***Area of residence***						
*Urban*						
*Rural*	*0.47 (0.27–0.82)*	*0.63 (0.36–1.12)*	*0.64 (0.36–1.13)*	***0.31 (0.15–0.66)***	***0.40 (0.19–0.85)***	***0.42 (0.20–0.90)***
*Moscow/St P*	***2.03 (1.32–3.12)***	*1.18 (0.73–1.91)*	*1.25 (0.77–2.04)*	*2.58 (1.69–3.92)*	*1.58 (0.98–2.56)*	*1.56 (0.96–2.55)*
***Employment status***						
*Working*	*1*	*1*	*1*	*1*	*1*	*1*
*Paid leave*	*0.93 (0.12–7.01)*	*0.72 (0.48–1.09)*	*0.53 (0.07–4.09)*	***2.06 (1.05–4.05)***	*1.90 (0.86–4.15)*	*2.24 (0.99–5.04)*
*Not working*	***0.12 (0.04–0.37)***	***0.58 (0.08–4.42)***	***0.13 (0.03–0.57)***	***0.31 (0.15–0.64)***	***0.38 (0.17–0.87)***	***0.35 (0.15–0.83)***
*Unemployed (sr)*	***0.13 (0.04–0.42)***	***0.15 (0.03–0.63)***	***0.24 (0.07–0.79)***	***0.08 (0.01–0.57)***	***0.13 (0.02–0.93)***	*0.14 (0.02–1.03)*
*Pension*	***0.09 (0.01–0.68)***	***0.27 (0.08–0.89)***	*0.14 (0.02–1.06)*	***0.06 (0.01–0.46)***	***0.12 (0.02–0.85)***	*0.11 (0.02–0.84)*
***Self-rated hlth (vg -5(vp)***	*0.90 (0.67–1.21)*	*1.04 (0.76–1.44)*	*1.11 (0.79–1.55)*	*0.87 (0.64–1.20)*	*1.06 (0.74–1.51)*	*1.14 (0.79–1.65)*

[Results in bold are statistically significant (p < 0.05)]

At the same time there was a very small absolute increase in the minority with supplementary (private) insurance from 2% to nearly 3%, which in three quarters of cases was paid for by their employer (Table 1). Amongst working age people there was little variation by age (Table 3). Those with supplementary insurance were more likely to be male, to be in employment, to have higher education, greater income and (in the case of women) more household assets, to live in Moscow or St Petersburg, and to be on paid leave (Table 3). Supplementary insurance was more common amongst married people than those who were single, widowed or cohabiting. In the fully adjusted model, employment status, urban/rural dwelling, household income (and, for men only, education) remained significant determinants of supplementary insurance.

The proportion of respondents reporting a recent health problem who chose to see a doctor (rather than self-treat) declined from 37% to 30% between 2000 and 2001, but remained stable thereafter (Table 1). Respondents without insurance were significantly less likely to seek help although, for men, this ceased to be significant once employment status was taken into account. (Table 4) Women with a health problem were slightly more likely to see a doctor than men, although this was accounted for by other factors. Although people with secondary education were less likely to seek professional help than those with only primary education, there was no association between professional help-seeking and higher education, except among women, in whom it ceased to be significant when adjusted for other factors. Single people and divorced men were significantly more likely to seek help than married respondents. Working age pensioners (who are likely to include those who retired early due to ill-health), and men who were otherwise not working (and not unemployed) were more likely to seek help.

**Table 4:** Determinants of (a) seeing a professional for a new health problem (b) using private healthcare-logistic regression (RLMS: men and women aged 18–59)

	**Male**			**Female**		
	**(i) Age, gender**	**(ii) Age, gend, inc, educ, area, marital**	**(iii) = (ii) + empl. status + s-r health**	**(i) Age, gender**	**(ii) Age, gend, inc, educ, area, marital**	**(iii) = (ii) + empl. status + s-r health**
	**SEEING A PROFESSIONAL (vs not seeing) – Odds ratio (95% CI)**					

**Sex (female vs male)**	-	-	-	**1.16 (1.01–1.33)**	1.17 (1.00–1.35)	1.03 (0.89–1.21)

**Age (per year)**	0.99 (0.98–1.00)	1.00 (0.99–1.02)	0.99 (0.98–1.00)	**1.00 (1.00–1.01)**	**1.01 (1.00–1.02)**	1.00 (0.99–1.01)
**Health ins.**						
Compulsory	1	1		1	1	
Suppl. (priv)	1.02 (0.53–1.97)	1.13 (0.56–2.27)	1.21 (0.60–2.45)	1.04 (0.63–1.70)	1.19 (0.69–2.07)	1.28 (0.73–2.23)
None	**0.62 (0.41–0.93)**	**0.64 (0.42–0.98)**	0.68 (0.44–1.06)	**0.56 (0.36–0.88)**	**0.53 (0.33–0.86)**	**0.56 (0.34–0.91)**
**Education**						
1ry/incomp 2ry	1	1		1	1	
Comp 2ry	**0.76 (0.58–0.99)**	0.79 (0.60–1.05)	0.89 (0.66–1.20)	**0.68 (0.54–0.87)**	**0.72 (0.56–0.93)**	0.77 (0.59–1.01)
Higher	0.82 (0.60–1.11)	0.91 (0.65–1.27)	1.11 (0.78–1.58)	**0.77 (0.61–0.97)**	0.85 (0.66–1.10)	0.95 (0.72–1.25)
**Hhld inc. pp quintile (asc.)**	0.96 (0.88–1.04)	0.96 (0.88–1.05)	1.01 (0.92–1.10)	0.98 (0.92–1.04)	0.98 (0.92–1.05)	1.00 (0.93–1.07)
**Nr consumer gds (0–5)**	0.92 (0.70–1.21)	0.96 (0.69–1.34)	1.03 (0.72–1.47)	0.97 (0.83–1.14)	0.91 (0.76–1.11)	0.87 (0.72–1.06)
**Marital status**						
Married	1	1		1	1	
Single	**1.63 (1.15–2.31)**	**1.56 (1.08–2.25)**	1.23 (0.81–1.86)	**1.49 (1.14–1.94)**	**1.46 (1.10–1.94)**	**1.44 (1.06–1.94)**
Divorced	**1.99 (1.24–3.19)**	**2.08 (1.27–3.41)**	1.67 (0.98–2.85)	0.92 (0.69–1.23)	0.92 (0.69–1.25)	0.97 (0.72–1.32)
Widowed	0.52 (0.15–1.80)	0.54 (0.15–1.89)	0.43 (0.12–1.55)	1.10 (0.79–1.54)	1.15 (0.81–1.62)	1.09 (0.77–1.56)
Cohabiting	1.15 (0.80–1.66)	1.15 (0.79–1.68)	1.24 (0.84–1.83)	1.10 (0.83–1.47)	1.09 (0.80–1.48)	1.09 (0.79–1.49)
**Geog. area**						
Urban	1	1		1	1	
Rural	0.96 (0.73–1.26)	0.93 (0.70–1.24)	1.00 (0.75–1.35)	1.05 (0.85–1.30)	1.03 (0.82–1.28)	1.06 (0.84–1.33)
Moscow/St P	1.18 (0.86–1.62)	1.18 (0.83–1.66)	1.22 (0.85–1.73)	1.12 (0.88–1.43)	1.18 (0.90–1.56)	1.22 (0.92–1.62)
**Employment**						
Working	1	1		1	1	
paid leave	1.09 (0.29–4.06)	1.14 (0.30–4.29)	1.00 (0.26–3.80)	1.27 (0.79–2.02)	1.46 (0.88–2.44)	1.55 (0.92–2.61)
Unpaid leave	-	-	-	3.13 (0.70–14.06)	2.84 (0.62–12.99)	2.96 (0.65–13.46)
not working	**1.74 (1.26–2.39)**	**1.71 (1.16–2.52)**	**1.57 (1.06–2.33)**	1.02 (0.80–1.31)	1.07 (0.81–1.42)	1.05 (0.79–1.40)
unemployed	0.95 (0.64–1.41)	0.94 (0.62–1.41)	0.92 (0.60–1.41)	**0.61 (0.42–0.90)**	**0.63 (0.42–0.94)**	**0.65 (0.43–0.97)**
Pension	**3.10 (2.07–4.62)**	**2.93 (1.89–4.55)**	**2.32 (1.47–3.66)**	**1.78 (1.35–2.35)**	**1.60 (1.18–2.17)**	1.33 (0.97–1.82)
**Self-rated hlth 1(vg) -5(vp)**	**1.81 (1.51–2.16)**	**1.78 (1.47–2.14)**	**1.65 (1.36–2.00)**	**1.98 (1.69–2.32)**	**2.02 (1.71–2.39)**	**2.00 (1.69–2.36)**

	**SEEKING PRIVATE HEALTH CARE (vs not seeking private care) – Odds ratio (95% CI)**					

**Sex (female vs male)**	-	-	-	**1.81 (1.15–2.85)**	1.52 (0.92– 2.52)	1.52 (0.92–2.53)

**Age (per year)**	0.99 (0.97–1.02)	0.98 (0.93–1.03)	1.00 (0.95–1.06)	0.98 (0.97–1.00)	0.96 (0.93–0.98)	0.96 (0.93–0.98)
**Health ins.**						
Compulsory	1	1	1	1	1	1
Suppl. (priv)	3.56 (0.72–17.58)	1.83 (0.28–12.08)	2.64 (0.40–17.26)	**3.03 (1.15–7.95)**	2.25 (0.72–7.00)	2.23 (0.71–6.98)
None	**4.61 (1.55–13.74)**	3.80 (0.96–15.08)	3.65 (0.85–15.71)	2.45 (0.87–6.93)	2.78 (0.81–9.51)	2.83 (0.81–9.81)
**Education**						
1ry/incomp 2ry	1	1	1	1	1	1
Comp 2ry	**4.94 (1.33–18.42)**	**7.06 (1.44–34.58)**	**8.28 (1.62–42.40)**	**2.68 (1.04–6.89)**	**3.24 (1.13–9.25)**	**3.26 (1.13–9.36)**
Higher	**8.59 (2.10–35.20)**	**9.14 (1.62–51.46)**	**12.06 (1.91–76.0)**	**6.14 (2.49–15.14)**	**6.52 (2.33–18.27)**	**6.21 (2.13–18.10)**
**Hhld inc. pp quintile (asc.)**	**1.74 (1.22–2.48)**	1.46 (0.99–2.17)	**1.49 (1.01–2.20)**	**1.23 (1.03–1.46)**	1.12 (0.93–1.36)	1.08 (0.89–1.32)
**Nr consumer gds (0–5)**	1.02 (0.94–1.10)	1.07 (0.97–1.18)	1.08 (0.98–1.19)	1.02 (0.96–1.09)	1.05 (0.97–1.13)	1.05 (0.97–1.13)
**Marital status**						
Married	1	1	1	1	1	1
Single	2.63 (0.71–9.74)	1.77 (0.48–6.52)	1.96 (0.43–8.88)	0.65 (0.33–1.29)	0.89 (0.43–1.85)	0.85 (0.40–1.82)
Divorced	0.63 (0.08–5.07)	0.81 (0.09–7.47)	0.97 (0.09–10.35)	0.82 (0.35–1.92)	0.86 (0.35–2.10)	0.80 (0.33–1.98)
Widowed	-	-	-	1.28 (0.52–3.13)	1.95 (0.75–5.09)	1.96 (0.75–5.13)
Cohabiting	1.30 (0.34–5.05)	0.64 (0.12–3.50)	0.81 (0.13–4.99)	0.40 (0.15–1.07)	0.20 (0.04–0.86)	0.19 (0.04–0.86)
**Geog. area**						
Urban	1	1	1	1	1	1
Rural	0.40 (0.09–1.81)	0.46 (0.10–2.19)	0.42 (0.08–2.08)	0.63 (0.33–1.23)	0.76 (0.38–1.52)	0.74 (0.37–1.49)
Moscow/St P	**4.21 (1.78–9.94)**	**2.88 (1.09–7.63)**	**2.87 (1.04–7.91)**	1.66 (0.92–2.97)	1.36 (0.67–2.76)	1.39 (0.68–2.82)
**Employment**						
Working	1	1	1	1	1	1
paid leave	7.00 (0.60–82.16)	14.32 (0.81–52.26)	14.32 (0.81–252.3)	0.35 (0.08–1.57)	0.45 (0.09–2.14)	0.45 (0.09–2.14)
Unpaid leave	-	-	-	-	-	-
not working	1.62 (0.53–4.97)	3.55 (1.00–12.69)	3.55 (1.00–12.69)	0.67 (0.34–1.32)	0.85 (0.39–1.88)	0.85 (0.39–1.88)
unemployed	1.47 (0.40–5.45)	3.49 (0.73–16.63)	3.49 (0.73–16.63)	0.52 (0.15–1.77)	0.59 (0.16–2.16)	0.59 (0.16–2.16)
Pension	0.28 (0.03–2.19)	-	-	0.38 (0.15–1.00)	0.72 (0.26–2.03)	0.72 (0.26–2.03)
**Self-rated hlth 1(vg)-5(vp)**	**0.42 (0.23–0.77)**	**0.41 (0.19–0.89)**	**0.37 (0.17–0.84)**	0.87 (0.59–1.28)	0.95 (0.62–1.46)	0.94 (0.61–1.45)

[Results in bold are statistically significant (p < 0.05)]

Amongst respondents who had sought professional help for a health problem within the last 30 days, use of the private sector rose significantly from 6% to 10% between 2000 and 2004 (Table 1). Women were almost twice as likely to use private health care as men (Table 4). Education was a very strong predictor, and partly explained the powerful effect of high income on private health care utilisation. Living in Moscow or St Petersburg was also a strong determinant in men. Men with poor health were significantly less likely to use private health care. As expected, the privately insured used private health care more often, but less expectedly, so did those without any form of health insurance. However, these associations were partly explained by socioeconomic factors.

## Discussion

### Summary of results

Compulsory health insurance coverage continued to expand in Russia between 2000 and 2004. The uninsured were typically poorer, less educated, unemployed and in worse health. Such individuals were also less likely to have private insurance. There was a small decline in those who sought medical help for a new health problem. The uninsured were less likely to seek medical help, independently of health and sociodemographic variables. Use of private healthcare increased during the study, although the proportion with private health insurance remained steady. Individuals using the private sector were more likely to be socioeconomically advantaged.

### Limitations

This study has several potential limitations. The most important relates to seeking professional medical assistance. It was not possible from these data to determine the nature of an individual's health problem, and thus the appropriateness of their decision to consult a healthcare professional or otherwise. Thus, some individuals may consult a professional inappropriately for minor illness, whilst others do not seek necessary help for more serious problems. Furthermore, this study does not differentiate between primary and secondary care use; in Bulgaria for example, those with lower socio-economic status would attend predominantly lower-quality primary care, but seek secondary level care less often. [20] Private health care and supplementary insurance were used by only a small, selected group, leading to wide confidence intervals that limited what could be concluded.

Interpretation of utilisation is also constrained by the known association of poor health with many of the socioeconomic factors being studied but by adjusting for subjective health it is possible to at least partly counteract the effects of selection, so that these data provide important insights into the relationship between health insurance and use of the health system in Russia.

In addition, these analyses did not take account either user fees (charged even to those with compulsory insurance) [21] or unofficial under the counter payments. [22] Both of these are important in Russia and other former Communist countries, and have the potential to influence health care utilisation but are difficult to research using survey data because of the problems of differentiating formal from informal payments, dealing with non-monetary gifts, and attributing some gifts (such as the expectation that future services will be provided by the patient) to a particular episode of care.

### Discussion of findings

On a positive note, the earlier rise in compulsory health insurance coverage continued, [12] and by 2004 most people were insured. Furthermore, the disadvantage of older people[12] had reversed, with coverage higher amongst people aged over 60 than in those of working age.

Nevertheless, it is concerning that by 2004 more than 10% of working age men had no insurance, and that uninsured individuals were multiply disadvantaged. As in 2000, unemployment strongly predicted non-insurance,[12] and coverage was lower in people living outside Moscow and St Petersburg.[12] In this analysis, asset score was better than income as a predictor of coverage. This is consistent with many surveys in this region, reflecting the limited ability of monetary income to capture economic status in a society where there is still extensive informal exchanges, including barter.[23] These findings demonstrate serious deficiencies in the way municipalities and other public entities cover unemployed people, a group that includes many who transition frequently in and out of the labour market. This contrasts with the more easily identifiable groups (those retired due to ill health or with consistently low income).

Importantly, respondents without mandatory insurance were less likely to consult a doctor for a health problem. This is likely to represent genuine under-utilisation, since it was not explained by poor subjective health. However, being in formal employment is also an important determinant of seeking health care, perhaps because some people access care through informal channels, or through occupational facilities or pharmacies, where the rules may not be strictly enforced.

In this study, the inconsistent associations between sociodemographic variables seeking professional help for a health problem contrasts with a previous study, where men, the socioeconomically disadvantaged and rural dwellers were less likely to seek help, although the inconsistencies may be explained by differences in the health care measure and the assessment of household resources. [4]

The greater likelihood of unemployed and pensioners to consult a professional, partly independent of subjective health, could reflect the reluctance of the full-time employed to endure long waiting times in the public sector. However, respondents reporting poorer health were independently more likely to seek health care, consistent with them consulting for more serious symptoms.

The relatively new phenomenon of private health insurance in Russia was taken up by a small, fairly constant minority. This contrasts with large increase in users of private health *care*, and could account for the relatively weak association between the two. However, the uninsured were also more likely to use private health care, suggesting that they may face obstacles in accessing the public sector, for example people without formal residence, migrants and temporary workers. In other countries undergoing transition, patients often resort to both public and private sector, in seeking access to particular specialists, better conditions of care and convenience. [20] Private and public services may occasionally be difficult to distinguish, when both are located within the same facility and require payment of a fee.

Availability of private health care provision will inevitably influence utilisation, which is likely to explain the greater use by men in Moscow and St Petersburg, and the slightly lower utilisation by rural dwellers. Fewer rural women had supplementary insurance.

Greater use of the private health care sector by the better educated and wealthier, and especially the former, suggests that more informed people may adopt a more strategic and consumerist approach to seeking health care, [15] although the quality of the private care obtained is clearly unknown. Elsewhere, this has been shown to be facilitated by the use of social networks.[2,15,16] A similar association was shown for people in better health. This may reflect differences in concepts and the value placed on good health, with the less educated perceiving health as a means to functioning at work, seeking care only when confronted with advanced illness, compared with the better educated, who regarded health as an asset for life, [15] and illustrates the complexity of the drivers of health care inequalities.

Relationships between gender and marital status and health care use were complex. Women were more likely to seek care for a health problem, consistent with previous research, [4] and to have compulsory insurance and use private health care. Whether this represents variations in health, or in healthcare seeking behaviour, is unclear. The greater likelihood of single (non-cohabiting) women to consult compared with married women, independently of socioeconomic and health status, is hard to explain. The tendency of married women to put their own needs second to those of their family provides a possible explanation.[24] The differences are unlikely to be due to reproductive patterns, since early marriage (or cohabitation) and first childbirth are still typical in Russia, [25] which also indicates that the gender differences in utilisation are unlikely to be due to differences in the need for reproductive care.

The associations between cohabiting, divorce, widowhood and being uninsured were stronger amongst men, and not fully explained by socioeconomic circumstances. In contrast, divorce and singleness predicted greater likelihood of professional consultation for a medical problem. Previous research has shown that social support (through formal and informal channels) is an important factor influencing the probability of seeking care. [3] Further research is required to examine the role of social support, or of valuing health, in the differences. However, it is particularly concerning that men in worse health were less likely to be insured, since premature male mortality and ill-health in working age men are major public health issues, and poor self-rated health predicts mortality in Russia [26] as elsewhere. [27]

## Conclusion

There are several reasons to believe that the 'inverse care law' [18] is operating in transitional Russia. Disadvantaged people are more likely to lack health insurance, and the uninsured themselves appear to be doubly disadvantaged. They are less likely to seek care for problems, despite experiencing worse health, and are also more likely to use private health services when they do need help.

Achieving universal insurance coverage is therefore an important step towards reducing inequitable access to health care. Furthermore, it may contribute to addressing avoidable healthcare-related mortality in Russia, although this complex area is subject to multiple influences including quality of care. [7] Further inequalities in care may result from the educated and healthy taking advantage of the growing private sector. Whilst health education is likely to be important, considerable further research is required to understand and address socioeconomic and gender differences in health care utilisation in Russia.

## Competing interests

The authors declare that they have no competing interests.

## Authors' contributions

FP developed the concept for the additional analyses of health care utilisation, analysed the data and drafted the paper. DB and MM developed the concept for the previous analyses of insurance use on which this paper builds, [12] redrafted the paper, and contributed to the discussion.

## Pre-publication history

The pre-publication history for this paper can be accessed here:



## References

[B1] Stuckler D, King L, McKee M (2009). Mass Privatization and the post-communist mortality crisis. Lancet.

[B2] Rusinova NL, Brown JV (1997). Russian medical care in the 1990s: A user's perspective. Social Science and Medicine.

[B3] Danishevski K, Balabanova D, McKee M, Atkinson S (2006). The fragmentary federation: experiences with the decentralized health system in Russia. Health Policy Plan.

[B4] Balabanova D, McKee M, Pomerleau J, Rose R, Haerpfer C (2004). Health service utilisation in the former Soviet Union: evidence from eight countries. Health Serv Res.

[B5] Hopkinson B, Balabanova D, McKee M, Kutzin J (2004). The human perspective on health care reform: coping with diabetes in Kyrgyzstan. Int J Health Planning Management.

[B6] Balabanova D, McKee M, Koroleva N, Chikovani I, Goguadze K, Kobaladze T, Adeyi O, Robles S (2009). Navigating the health system: diabetes care in Georgia. Health Policy and Planning.

[B7] Andreev E, Nolte E, Shkolnikov V, Varavikova EA, McKee M (2003). The evolving pattern of avoidable mortality in Russia. International Journal of Epidemiology.

[B8] Tragakes E, Lessof S (2003). Health care systems in transition: Russian Federation.

[B9] Twigg JL (1999). Regional variation in Russian medical insurance: lessons from Moscow and Nizhny Novgorod. Health Place.

[B10] Twigg JL (2002). Health care reform in Russia: a survey of head doctors and insurance administrators. Soc Sci Med.

[B11] Burger EJJ, Field MG, Twigg JL (1998). From assurance to insurance in Russian health care: the problematic transition. Am J Public Health.

[B12] Balabanova D, Falkingham J, McKee M (2003). Winners and losers: expansion of insurance coverage in Russia in the 1990s. American Journal of Public Health.

[B13] Rese A, Balabanova D, Danishevski K, McKee M, Sheaff R (2005). Implementing general practice in Russia: getting beyond the first steps. British Medical Journal.

[B14] Perlman F, McKee M (2007). Diabetes during the Russian transition. Diabetes Research and Clinical Practice.

[B15] Rusinova NL, Brown JV (2003). Social inequality and strategies for getting medical care in post-Soviet Russia. Health (London).

[B16] Salmi AM (2003). Health in exchange: teachers, doctors, and the strength of informal practices in Russia. Culture, Medicine and Psychiatry.

[B17] Marquez P (2008). Public spending in Russia for health care: issues and options.

[B18] Hart JT (1971). The inverse care law. Lancet.

[B19] Atkinson A, Rainwater L, Smeeding T (1995). Income distribution in OECD countries: the evidence from the Luxembourg Income Study (LIS).

[B20] Balabanova D, McKee M (2002). Access to health care in a system transition: the case of Bulgaria. Int J Health Plann Mgnt.

[B21] Larivaara M, Dubikaytis T, Kuznetsova O, Hemminki E (2008). Between a rock and a hard place: the question of money at St. Petersburg women's clinics. International Journal of Health Services.

[B22] Balabanova D, McKee M (2002). Understanding informal payments for health care: the example of Bulgaria. Health Policy.

[B23] Ledeneva A (1998). Russia's economy of favours: Blat, networking and social exchange.

[B24] Maltby H (1999). The common thread: health care activities of Vietnamese and Anglo-Australian women. Health Care for Women International.

[B25] Avdeev A, Monnier A (1995). A Survey of Modern Russian Fertility. Population: An English Selection.

[B26] Perlman F, Bobak M (2008). Determinants of self rated health and mortality in Russia – are they the same?. Int J Equity Health.

[B27] Idler E, Benyamini Y (1997). Self-rated health and mortality: a review of twenty-seven community studies. Journal of Health and Social Behavior.

